# Involvement of TRPM2 in Peripheral Nerve Injury-Induced Infiltration of Peripheral Immune Cells into the Spinal Cord in Mouse Neuropathic Pain Model

**DOI:** 10.1371/journal.pone.0066410

**Published:** 2013-07-30

**Authors:** Kouichi Isami, Kayo Haraguchi, Kanako So, Kayoko Asakura, Hisashi Shirakawa, Yasuo Mori, Takayuki Nakagawa, Shuji Kaneko

**Affiliations:** 1 Department of Molecular Pharmacology, Graduate School of Pharmaceutical Sciences, Kyoto University, Kyoto, Japan; 2 Department of Synthetic Chemistry and Biological Chemistry, Graduate School of Engineering, Kyoto University, Kyoto, Japan; Hokkaido University, Japan

## Abstract

Recent evidence suggests that transient receptor potential melastatin 2 (TRPM2) expressed in immune cells plays an important role in immune and inflammatory responses. We recently reported that TRPM2 expressed in macrophages and spinal microglia contributes to the pathogenesis of inflammatory and neuropathic pain aggravating peripheral and central pronociceptive inflammatory responses in mice. To further elucidate the contribution of TRPM2 expressed by peripheral immune cells to neuropathic pain, we examined the development of peripheral nerve injury-induced neuropathic pain and the infiltration of immune cells (particularly macrophages) into the injured nerve and spinal cord by using bone marrow (BM) chimeric mice by crossing wildtype (WT) and TRPM2-knockout (TRPM2-KO) mice. Four types of BM chimeric mice were prepared, in which irradiated WT or TRPM2-KO recipient mice were transplanted with either WT-or TRPM2-KO donor mouse-derived green fluorescence protein-positive (GFP^+^) BM cells (TRPM2^BM+/Rec+^, TRPM2^BM–/Rec+^, TRPM2^BM+/Rec–^, and TRPM2^BM–/Rec–^ mice). Mechanical allodynia induced by partial sciatic nerve ligation observed in TRPM2^BM+/Rec+^ mice was attenuated in TRPM2^BM–/Rec+^, TRPM2^BM+/Rec–^, and TRPM2^BM–/Rec–^ mice. The numbers of GFP^+^ BM-derived cells and Iba1/GFP double-positive macrophages in the injured sciatic nerve did not differ among chimeric mice 14 days after the nerve injury. In the spinal cord, the number of GFP^+^ BM-derived cells, particularly GFP/Iba1 double-positive macrophages, was significantly decreased in the three TRPM2-KO chimeric mouse groups compared with TRPM2^BM+/Rec+^ mice. However, the numbers of GFP^–^/Iba1^+^ resident microglia did not differ among chimeric mice. These results suggest that TRPM2 plays an important role in the infiltration of peripheral immune cells, particularly macrophages, into the spinal cord, rather than the infiltration of peripheral immune cells into the injured nerves and activation of spinal-resident microglia. The spinal infiltration of macrophages mediated by TRPM2 may contribute to the pathogenesis of neuropathic pain.

## Introduction

Neuropathic pain is a pathological pain condition that often results from peripheral nerve injury. Several lines of evidence suggest that neuroinflammation mediated by the interaction between immune cells and neurons plays an important role in neuropathic pain [1], [2]. In response to peripheral nerve injury, peripheral immune cells, such as macrophages, neutrophils, T-lymphocytes and mast cells, infiltrate into the injured nerve and become activated. Pronociceptive inflammatory mediators released from the activated immune cells can induce the sensitization of nociceptors and increase the excitability of nociceptive primary afferent neurons (peripheral sensitization). In the spinal cord, glial cells such as microglia and astrocytes receive signals from the injured peripheral neurons and become activated, which cause the generation of synaptic facilitation and enhanced responsiveness of nociceptive dorsal horn neurons (central sensitization) [3]. Additionally, there is increasing evidence that peripheral nerve injury induces the infiltration of peripheral immune cells into the spinal cord, which contributes to the pathogenesis of neuropathic pain [4]–[8], although the details and mechanisms still remain unclear.

Transient receptor potential melastatin 2 (TRPM2), a nonselective cation channel, acts as a sensor for reactive oxygen species (ROS) [9], [10]. TRPM2 is highly expressed in the brain and broadly in other tissues [11], [12], but is also expressed abundantly in immune cells, including monocytes/macrophages, neutrophils, T-lymphocytes and microglia [13]–[15]. Recent studies have focused extensively on the roles of TRPM2 expressed in immune cells. TRPM2-mediated Ca^2+^ influx in monocytes induces the production of proinflammatory cytokines/chemokines and the infiltration of neutrophils, which contribute to the exacerbation of inflammation [16]–[18]. TRPM2 expressed in T-lymphocytes contributes to the cell proliferation and the production/release of proinflammatory cytokines [19], [20]. In dendritic cells, TRPM2 acts a lysosomal Ca^2+^-release channel that promotes chemokine responsiveness and cell migration [21]. By contrast, TRPM2-knockout (TRPM2-KO) mice are susceptible to bacterial infection due to impaired inflammatory responses and uncontrolled bacterial growth [22]. In this context, TRPM2 in phagocytic cells prevents nicotinamide adenine dinucleotide phosphate oxidase-derived ROS production through depolarization of the plasma membrane and thereby protects against inflammation and tissue injury [23]. Taken together, these findings suggest that TRPM2 expressed in immune cells plays a critical role in immune and inflammatory responses.

Recently, we reported that TRPM2 expressed in macrophages and spinal microglia contributes to the pathogenesis of inflammatory and neuropathic pain through the aggravation of peripheral and central pronociceptive inflammatory responses in mice [24]. However, previous experiments using TRPM2-KO mice did not determine whether TRPM2 expressed in peripheral immune cells or spinal microglia is more relevant to neuropathic pain. The present study further explored the role of TRPM2 expressed in peripheral immune cells in neuropathic pain by generating bone marrow (BM) chimeric mice by crossing wildtype (WT) and TRPM2-KO mice with green fluorescence protein-positive (GFP^+^) BM transplantation. We then examined the development of peripheral nerve injury-induced neuropathic pain, and observed Iba1-positive (Iba1^+^) macrophages/microglia and GFP^+^ BM-derived cells in the injured sciatic nerve and spinal cord.

## Materials and Methods

### Animals

This study was carried out in strict accordance with the recommendations in the Guiding Principles for the Care and Use of The Japanese Pharmacological Society. The protocol was approved by the Kyoto University Animal Research Committee (Permit Number: 2012–24 and 2013–24). All efforts were made to minimize the number of animals used and to limit experimentation to that necessary to produce reliable scientific information. TRPM2-KO mice were generated as previously reported [16]. The TRPM2-KO mouse line was backcrossed with C57BL/6J mice for ten generations to eliminate any background effects on the phenotype. C57BL/6-Tg(CAG-EGFP)C14–Y01-FM131Osb transgenic mice (GFP-transgenic mice), a transgenic line with an EGFP cDNA under the control of a chicken β-actin promoter and cytomegalovirus enhancer [25], and C57BL/6J mice were purchased from Nihon SLC (Shizuoka, Japan). All animals were group-housed with free access to food and water and maintained on a 12-h light/dark cycle.

### Generation of BM Chimeric Mice

Homozygous TRPM2^−/−^male mice were crossed with GFP-transgenic female mice to produce GFP^+^ TRPM2^+/−^mice. GFP^+^ TRPM2^+/+^ (WT) mice and GFP^+^ TRPM2^−/−^ (TRPM2-KO) mice were obtained by the hetero-mating of GFP^+^ TRPM2^+/−^female and male mice to obtain suitable BM donor mice. BM transplantation was carried out as previously reported [26] with slight modifications. BM recipients were male 6-week-old C57BL/6J or TRPM2-KO mice. Recipient mice were lethally irradiated with 10 Gy total body irradiation for 10 min. GFP^+^ WT or TRPM2-KO donor mice were euthanized by decapitation, their femurs were isolated, and both ends were cut and placed into a microtube. The femurs were centrifuged at 2000 rpm for 10 min, and the pellet of GFP^+^ BM cells was suspended in sterile phosphate-buffered saline (PBS). Between 3–5 h after the irradiation, the WT or TRPM2-KO recipient mice were transplanted with 4.0×10^6^ BM cells by an intravenous injection into the tail vein. WT recipient mice transplanted with WT donor mouse-derived GFP^+^ BM cells (TRPM2^BM+/Rec+^), WT recipient mice transplanted with TRPM2-KO donor mouse-derived GFP^+^ BM cells (TRPM2^BM–/Rec+^), TRPM2-KO recipient mice transplanted with WT donor mouse-derived GFP^+^ BM cells (TRPM2^BM+/Rec–^), and TRPM2-KO recipient mice transplanted with TRPM2-KO donor mouse-derived GFP^+^ BM cells (TRPM2^BM–/Rec–^) were housed in an environment of specific pathogen-free conditions with free access to autoclaved pellets and kanamycin-containing autoclaved water (1∶10,000). After 6 weeks, all chimeric animals were housed in a conventional environment, and male BM chimeric mice were used for pSNL surgery at the age of 12 weeks.

### Flow Cytometry

Flow cytometry was used to identify the purity of GFP^+^ cells in the blood after the BM transplantation. Peripheral blood (100 µl) was collected from the tail vein of each chimeric mouse at 6 weeks after BM transplantation. Collected blood was dissolved in 300 µl of saline diluted three times for approximately 10 s to hemolyze the erythrocytes. After adding 1000 µl of saline to restore the osmotic pressure, the solution was centrifuged for 5 min at 2,000×*g*. After pipetting off the supernatant, the cells were washed by adding 1000 µl of saline and centrifuged for 5 min at 2,000×*g*. After pipetting off the supernatant, 500 µl of fluorescence-activated cell sorting (FACS) buffer (0.02 M ethylenediaminetetraacetic acid and 0.01% bovine serum albumin in PBS) was added. The purity of GFP^+^ cells was assessed by FACS (Gallios, Beckman Coulter, Brea, California).

### Neuropathic Pain Model

For the pSNL model of neuropathic pain, surgery was performed as previously described, with slight modifications [27], [28]. Briefly, under sodium pentobarbital anesthesia, a 5 mm incision was made and the right sciatic nerve was exposed just distal to the branch leading to the posterior biceps femoris/semitendinous muscles. One-third to one-half of the diameter of the right sciatic nerve at the upper thigh level was ligated tightly with a 9-0 silk suture. The wound was closed by suturing the muscle and skin layers.

### Behavioral Test

Animals were acclimatized to the testing environment for at least 1 h before the behavioral test. The same experimenter handled and tested animals in the experiment and was blinded to the genotype of each animal. Mechanical sensitivity was assessed by measuring the paw withdrawal threshold using calibrated von Frey filaments as previously described, with slight modifications [29], [30]. Mice were acclimatized on a metal mesh floor in small cylinders for 1 h. Mechanical sensitivity was evaluated using a set of seven calibrated von Frey filaments (0.008, 0.02, 0.04, 0.07, 0.16, 0.4, and 1.0 g) that were applied to the plantar surface of the hindpaw until the filament bent slightly for a few seconds. The first stimulus was always the 0.16 g filament. When a mouse demonstrated a positive response such as flicking and lifting, the next lower-weight filament was applied. When a mouse demonstrated a negative response (i.e., no movement) the next higher-weight filament was applied. After the first change in response, four additional responses were observed, and the 50% paw withdrawal threshold was calculated [29], [31].

### Immunohistochemistry

Mice were deeply anesthetized with sodium pentobarbital and perfused through the ascending aorta with PBS followed by 4% (W/V) paraformaldehyde in phosphate buffer. The sciatic nerve was cut 5 mm on either side of the ligation site, and the L3–L5 spinal cord was extirpated from pSNL-induced neuropathic pain model mice. Then samples were postfixed for 4 h and cryoprotected overnight at 4°C in 15% sucrose. The tissues were frozen and sectioned with a cryostat (Leica, Nussloch, Germany). The sections (20 µm) were treated with 4% normal goat serum for 1 h at room temperature. After washing with PBS, the sections were incubated with a primary antibody directed against Iba-1 (rabbit anti-Iba-1 antibody, 1∶500; Wako Pure Chemical Industries, Osaka, Japan) at 4°C overnight. The sections were washed three times in PBS and labeled with fluorescence-labeled secondary antibody (Alexa Fluor 594-labeled goat anti-rabbit IgG, 1∶500; Molecular Probes, Invitrogen, Life Technologies, Carlsbad, CA) at room temperature for 1 h in the dark. After washing three times in PBS, the sections were mounted in the anti-fading medium Vectashield (Vector Laboratories, Burlingame, CA). Five non-adjacent sections of the sciatic nerve and the L3–L5 spinal cord were randomly selected for each animal. Confocal fluorescence images were observed using a confocal laser scanning microscope (Fluoview FV10i system, Olympus, Tokyo, Japan). In each section of the sciatic nerve, a fluorescence image (200×200 µm) was captured in the area approximately 500 µm distant from the ligation site. In each section of the spinal cord, a fluorescence image (300×200 µm) of contralateral and ipsilateral spinal dorsal horn was captured in the area. Iba1^+^ and GFP^+^ cells were counted in contralateral and ipsilateral regions of interest with Image J software (National Institute of Mental Health, Bethesda, MD). Three to six mice were included in each group.

### Statistical Analysis

Data are presented as the mean ± SEM and were analyzed using GraphPad Prism version 5.0. Statistical analyses of the 50% withdrawal thresholds were performed using the Kruskal-Wallis test followed by *post hoc* Dunn’s comparison test at each time point and each BM chimeric mouse group, respectively. The numbers of Iba1^+^ or GFP^+^ cells were analyzed by one-way analysis of variance (ANOVA) followed by *post hoc* Tukey-Kramer comparison test. In all cases, differences of *p*<0.05 were considered statistically significant.

## Results

### Neuropathic Pain in BM Chimeric mice

**Figure 1:**
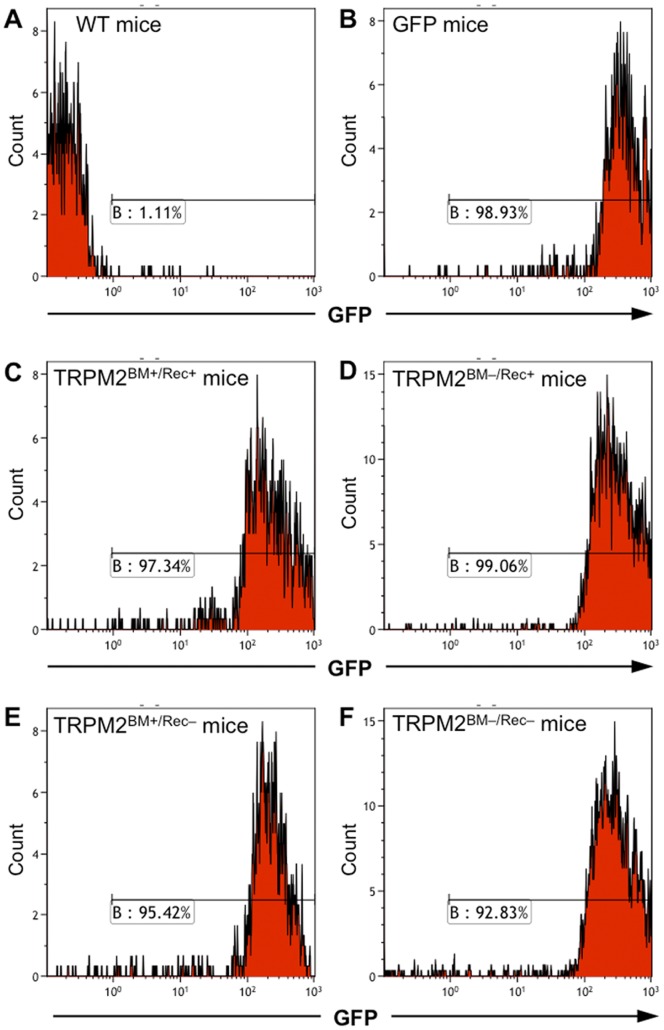
Flow cytometry analysis of BM-derived cells in WT/TRPM2-KO BM chimeric mice. Representative histograms of GFP^+^ cells in WT mice (A; negative control), GFP-transgenic mice (B; positive control), TRPM2^BM+/Rec+^ mice (C), TRPM2^BM–/Rec+^ mice (D), TRPM2^BM+/Rec–^ mice (E), and TRPM2^BM-/Rec–^ mice (F). In all examined chimeric mice, more than 90% of the BM-derived cells were GFP^+^.

**Figure 2:**
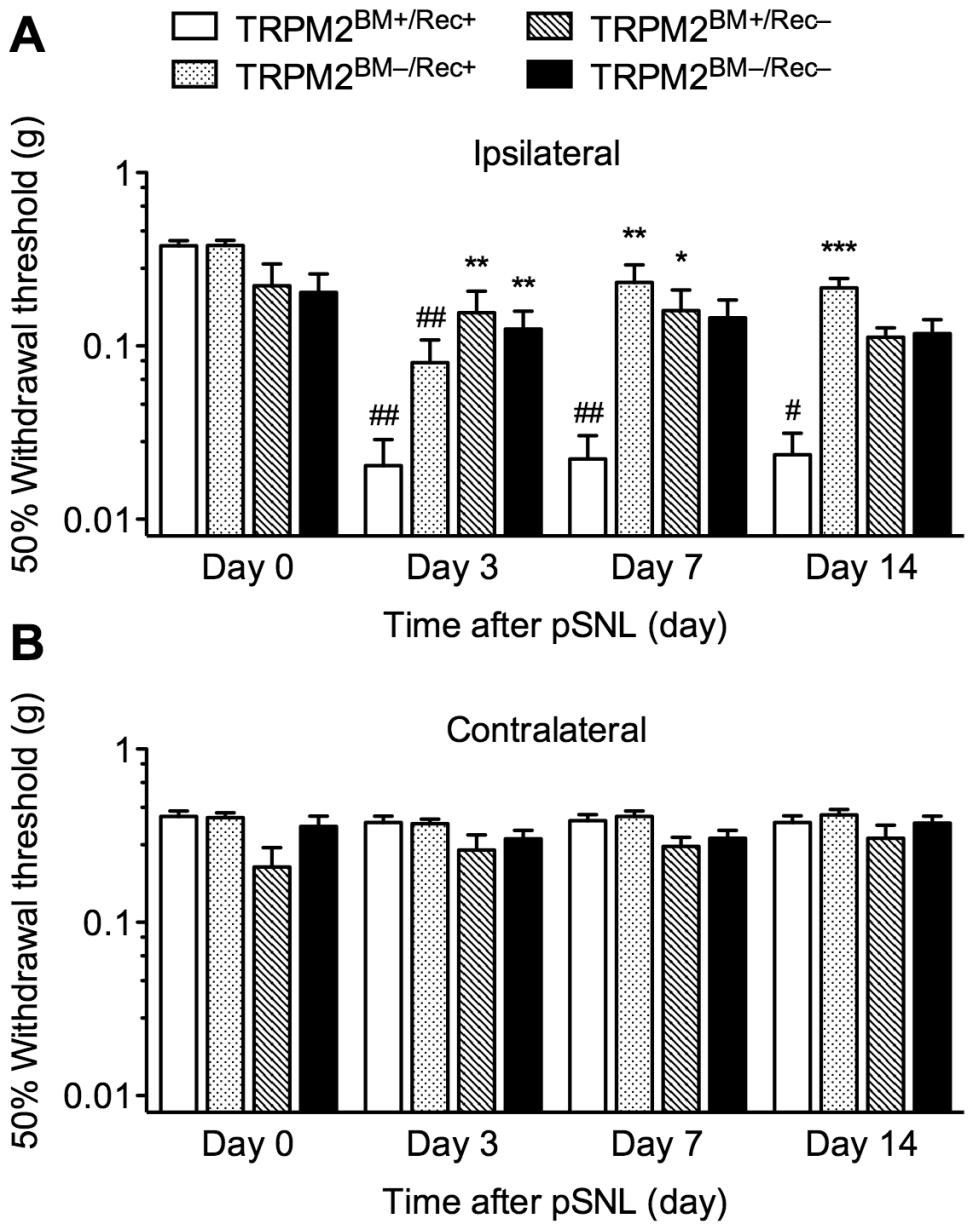
Peripheral nerve injury-induced mechanical allodynia in WT/TRPM2-KO BM chimeric mice. In the pSNL model of neuropathic pain, the 50% withdrawal threshold to mechanical stimuli was determined in the ipsilateral (**A**) and contralateral paws (**B**) of TRPM2^BM+/Rec+^, TRPM2^BM–/Rec+^, TRPM2^BM+/Rec–^, and TRPM2^BM+/Rec+^ mice. *p < 0.05; **p < 0.01; ***p < 0.001, compared with TRPM2^BM+/Rec+^ mice. ^#^ *p* <0.05;^ ##^ *p* <0.01, compared with Day 0 in each BM chimeric mouse group. *n* = 5–7. Data are expressed as the mean ± SEM.

To determine whether TRPM2 expressed in peripheral immune cells contributes to neuropathic pain, irradiated WT or TRPM2-KO recipient mice were transplanted with either WT-or TRPM2-KO donor mouse-derived GFP^+^ BM cells to generate four types of BM chimeric mice (TRPM2^BM+/Rec+^, TRPM2^BM–/Rec+^, TRPM2^BM+/Rec–^, TRPM2^BM–/Rec–^ mice) (see Materials and Methods for details). Flow cytometric analysis revealed that, 6 weeks after BM transplantation, more than 90% of the BM-derived cells in the blood of the chimeric mice were replaced with GFP^+^ cells (Fig. 1). In TRPM2^BM+/Rec+^ mice, partial sciatic nerve ligation (pSNL) surgery significantly decreased the 50% withdrawal threshold to mechanical stimulation in the ipsilateral paw (*p*<0.001, Kruskal-Wallis test). Significant decreases were observed 3, 7 and 14 days after pSNL surgery, compared with pre-surgery (Day 0). In TRPM2^BM–/Rec+^, TRPM2^BM+/Rec–^, and TRPM2^BM–/Rec–^ mice, pSNL-induced mechanical allodynia was attenuated. In TRPM2^BM–/Rec+^ mice, although pSNL surgery significantly decreased the 50% withdrawal threshold in the ipsilateral paw (*p*<0.001, Kruskal-Wallis test), a significant decrease was observed only 3 days, but not 7 and 14 days, after pSNL surgery, compared with pre-surgery (Day 0). Compared with TRPM2^BM+/Rec+^ mice, pSNL-induced mechanical allodynia was significantly attenuated on Day 7 and 14. In TRPM2^BM+/Rec–^ and TRPM2^BM–/Rec–^ mice, pSNL surgery had no effect on the 50% withdrawal threshold in the ipsilateral paw, and no significant mechanical allodynia was observed during the observed period, compared with pre-surgery (Day 0). Compared with TRPM2^BM+/Rec+^ mice, pSNL-induced mechanical allodynia was significantly attenuated on Day 3 and 7 in TRPM2^BM+/Rec–^ mice, and on Day 3 in TRPM2^BM–/Rec–^ mice. On the other hand, the 50% withdrawal thresholds in the contralateral paws were not changed in any chimeric mice (Fig. 2).

### Iba1^+^ and GFP^+^ Cells in the Injured Sciatic Nerve in BM Chimeric mice

**Figure 3:**
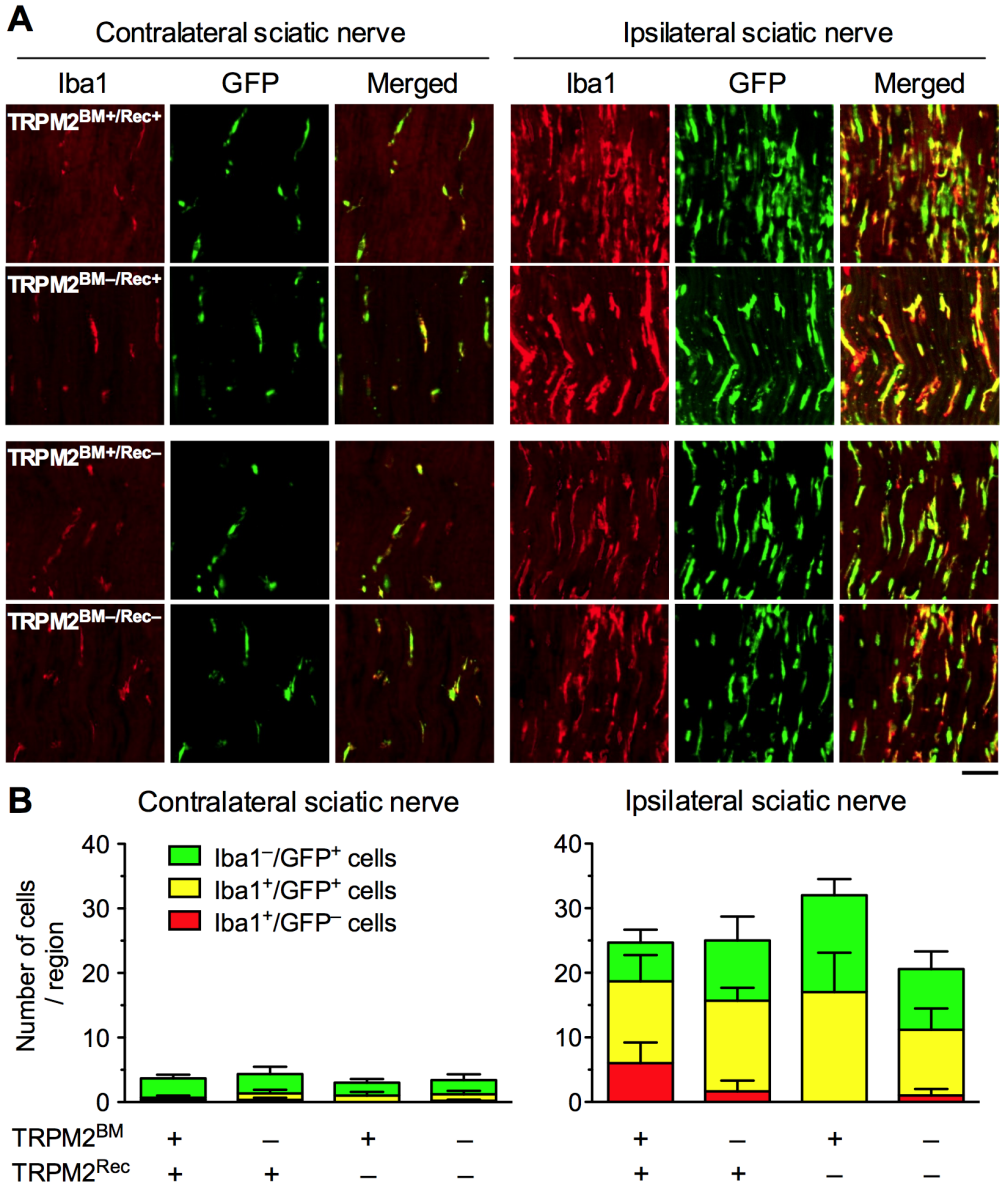
Infiltration of GFP^+^ BM-derived cells around the injured sciatic nerve in WT/TRPM2-KO BM chimeric mice. (**A**) GFP^+^ cells and Iba1^+^ cells were visualized by GFP fluorescence (green) and immunostaining with Iba1 antibody (red), respectively, in the sciatic nerve sections around the ligation site 14 days after pSNL surgery. Iba1^+^/GFP^+^ cells were visualized as a yellow signal in merged images. Representative microphotographs are shown (scale bars = 50 µm). (**B**) The numbers of Iba1^−^/GFP^+^ cells, Iba1^+^/GFP^−^cells, and Iba1^+^/GFP^+^ cells within the images were counted in the contralateral (left panel) and ipsilateral (right panel) sections. *n* = 3–5. Data are expressed as the mean ± SEM.

Our preliminary data using GFP^+^ BM chimeric mice showed that peripheral nerve injury induced infiltration of GFP^+^ BM-derived cells in the ipsilateral peripheral nerve, which gradually increased between Day 3 and 14. On Day 14, the infiltration of GFP^+^ BM-derived cells was maximal, and approximately half of them were Iba1^+^ macrophages. In this study, we identified Iba1^+^ and GFP^+^ cells in the injured sciatic nerve 14 days after pSNL surgery in TRPM2^BM+/Rec+^, TRPM2^BM–/Rec+^, TRPM2^BM+/Rec–^, TRPM2^BM–/Rec–^ mice (Fig. 3, Table 1). Small numbers of Iba1^+^ cells and GFP^+^ cells were observed in the contralateral sciatic nerve in all chimeric mice. Some GFP^+^ cells were double positive for Iba1. No differences were observed in the number of Iba1^+^ cells (*F*_3,10_ = 0.22, *p* = 0.881) and GFP^+^ cells (*F*_3,10_ = 0.20, *p* = 0.896) among chimera groups. Merged images revealed no difference in the number of Iba1^–^/GFP^+^ cells (*F*_3,10_ = 0.31, *p* = 0.816) or Iba1^+^/GFP^+^ cells (*F*_3,10_ = 0.33, *p* = 0.806) among chimera groups. Few Iba1^+^/GFP^–^ cells were observed in any of the chimera groups, and no difference was observed among chimera groups (*F*_3,10_ = 0.35, *p* = 0.790).

**Table 1:** The numbers of Iba1^+^ and GFP^+^ cells around the injured sciatic nerve.

	Contralateral				Ipsilateral			
	TRPM2^BM+/Rec+^	TRPM2^BM–/Rec+^	TRPM2^BM+/Rec–^	TRPM2^BM–/Rec–^	TRPM2^BM+/Rec+^	TRPM2^BM–/Rec+^	TRPM2^BM+/Rec–^	TRPM2^BM–/Rec–^
Total cells	3.7 ± 0.7	4.3 ± 1.3	3.0 ± 0.6	3.4 ± 0.8	24.7 ± 7.3	25.0 ± 0.6	32.0 ± 4.6	20.6 ± 3.0
Iba1^+^ cells	0.7 ± 0.3	1.3 ± 0.6	1.0 ± 0.6	1.2 ± 0.6	18.7 ± 7.3	15.7 ± 3.7	17.0 ± 6.1	11.2 ± 3.6
GFP^+^ cells	3.3 ± 0.9	4.0 ± 1.5	3.0 ± 0.6	3.2 ± 0.7	18.7 ± 4.3	23.3 ± 1.8	32.0 ± 4.6	19.6 ± 2.8
Iba1^–^/GFP^+^ cells	3.0 ± 0.6	3.0 ± 1.2	2.0 ± 0.6	2.2 ± 0.9	6.0 ± 2.0	9.3 ± 3.7	15.0 ± 2.5	9.4 ± 2.7
Iba1^+^/GFP^–^ cells	0.3 ± 0.3	0.3 ± 0.3	0	0.2 ± 0.2	6.0 ± 3.2	1.7 ± 1.7	0	1.0 ± 1.0
Iba1^+^/GFP^+^ cells	0.3 ± 0.3	1.0 ± 0.6	1.0 ± 0.6	1.0 ± 0.5	12.7 ± 4.1	14.0 ± 2.0	17.0 ± 6.1	10.2 ± 3.3

The numbers of Iba1^+^ cells, GFP^+^ cells, Iba1^+^/GFP^–^ cells, Iba1^–^/GFP^+^ cells, and Iba1^+^/GFP^+^ cells were counted in the contralateral and ipsilateral sciatic nerve 14 days after pSNL surgery. “Total cells” indicates the sum of Iba1^+^/GFP^–^ cells, Iba1^–^/GFP^+^ cells, and Iba1^+^/GFP^+^ cells. *n* = 3–5. Data are expressed as mean ± SEM.

In the ipsilateral sciatic nerve, Iba1^+^ cells and GFP^+^ cells were dramatically increased, and 50–65% of GFP^+^ cells were positive for Iba1. Although the number of GFP^+^ cells tended to increase in TRPM2^BM+/Rec–^ mice and decrease in TRPM2^BM–/Rec–^ mice, no significant differences were observed in the numbers of Iba1^+^ cells (*F*_3,10_ = 0.48, *p* = 0.706) and GFP^+^ cells (*F*_3,10_ = 2.93, *p* = 0.086) among chimera groups. Furthermore, merged photographs revealed no differences in the numbers of Iba1^–^/GFP^+^ cells (*F*_3,10_ = 1.43, *p* = 0.292) or Iba1^+^/GFP^+^ cells (*F*_3,10_ = 0.53, *p* = 0.670) among chimera groups. The number of Iba1^+^/GFP^–^ cells was low in all chimera groups, and no difference was observed among chimera groups (*F*_3,10_ = 2.21, *p* = 0.150).

### Iba1^+^ and GFP^+^ Cells in the Spinal Dorsal Horn in Chimeric mice

**Figure 4:**
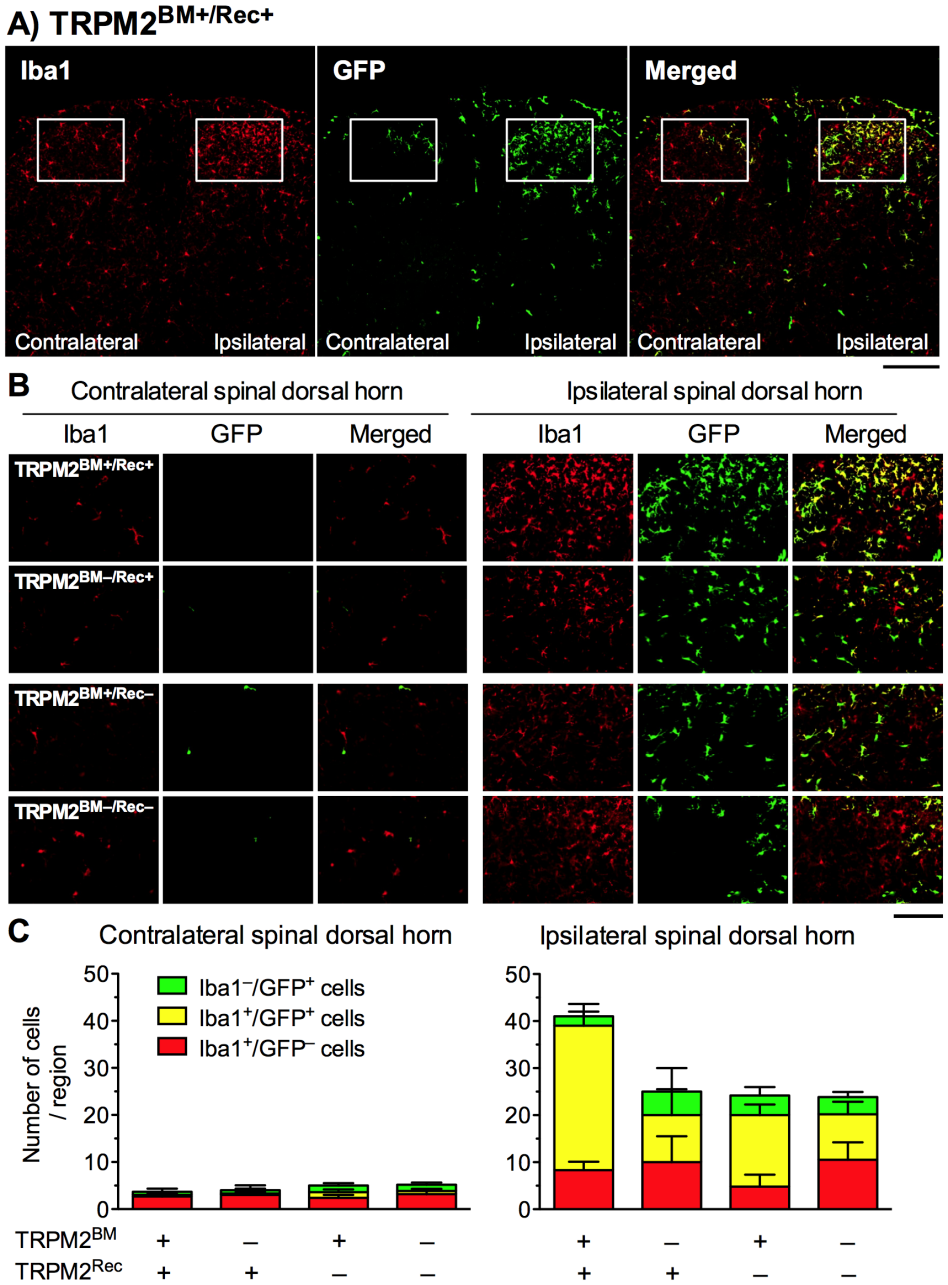
Infiltration of GFP^+^ BM-derived cells into the spinal cord in WT/TRPM2-KO BM chimeric mice. (**A, B**) GFP^+^ cells and Iba1^+^ cells were visualized by GFP fluorescence (green) and immunostaining with Iba1 antibody (red), respectively, in the spinal cord sections 14 days after pSNL surgery. Iba1^+^/GFP^+^ cells were visualized as a yellow signal in merged images. (**A**) Representative microphotographs in WT-BM and WT chimeric mice are shown (scale bars = 200 µm). (**B**) Representative microphotographs in selected regions of contralateral and ipsilateral spinal dorsal horn (defined by the rectangular area in A) are shown (scale bars = 100 µm). (**C**) The numbers of Iba1^-^/GFP^+^ cells, Iba1^+^/GFP^-^cells, and Iba1^+^/GFP^+^ cells within the selected regions were counted in the contralateral (left panel) and ipsilateral (right panel) spinal dorsal horn. *n* = 3–6. Data are expressed as the mean ± SEM.

Our preliminary data using GFP^+^ BM chimeric mice showed that peripheral nerve injury induced infiltration of GFP^+^ BM-derived cells into the ipsilateral spinal dorsal horn, which peaked between Day 7 and 14. Other previous findings have also shown that spinal infiltration of peripheral immune cells is maximal between 7 and 21 days after the peripheral nerve injury [4], [6], [8]. In this study, we identified Iba1^+^ cells and GFP^+^ cells in the spinal dorsal horn 14 days after pSNL surgery in TRPM2^BM+/Rec+^, TRPM2^BM–/Rec+^, TRPM2^BM+/Rec–^, TRPM2^BM–/Rec–^ mice (Fig. 4, Table 2). In the contralateral dorsal horn, a few Iba1^+^ cells were observed, but there were no or few GFP^+^ cells or Iba1^+^/GFP^+^ cells in any of the chimeric mice. No differences were observed in the numbers of Iba1^+^ cells (*F*_3,13_ = 0.07, *p* = 0.976) and GFP^+^ cells (*F*_3,13_ = 1.08, *p* = 0.392). Merged photographs revealed that no differences in the numbers of Iba1^+^/GFP^–^ cells (*F*_3,13_ = 0.11, *p* = 0.954), Iba1^–^/GFP^+^ cells (*F*_3,13_ = 0.52, *p* = 0.677) and Iba1^+^/GFP^+^ cells (*F*_3,13_ = 0.58, *p* = 0.640) among chimera groups.

**Table 2:** The numbers of Iba1^+^ and GFP^+^ cells in the spinal dorsal horn.

	Contralateral				Ipsilateral			
Donor (BM)	TRPM2^BM+/Rec+^	TRPM2^BM–/Rec+^	TRPM2^BM+/Rec–^	TRPM2^BM–/Rec–^	TRPM2^BM+/Rec+^	TRPM2^BM–/Rec+^	TRPM2^BM+/Rec–^	TRPM2^BM–/Rec–^
Total cells	3.7 ± 0.7	4.0 ± 2.0	5.0 ± 0.4	5.2 ± 1.7	41.0 ± 4.6	25.0 ± 10.1	24.3 ± 1.5	23.8 ± 5.8
Iba1^+^ cells	3.0 ± 0.0	3.3 ± 1.9	3.6 ± 0.9	3.8 ± 1.4	39.0 ± 3.6	20.0 ± 7.9	20.0 ± 2.0	20.2 ± 5.7
GFP^+^ cells	1.0 ± 1.0	1.0 ± 0.6	2.6 ± 0.2	2.0 ± 0.8	32.7 ± 5.5	15.0 ± 10.0	19.4 ± 3.2	13.3 ± 2.6
Iba1^–^/GFP^+^ cells	0.7 ± 0.7	0.7 ± 0.3	1.4 ± 0.5	1.3 ± 0.5	2.0 ± 1.0	5.0 ± 5.0	4.2 ± 1.8	3.7 ± 1.1
Iba1^+^/GFP^–^ cells	2.7 ± 0.3	3.0 ± 2.1	2.4 ± 0.6	3.2 ± 1.1	8.3 ± 1.8	10.0 ± 5.5	4.8 ± 2.6	10.5 ± 3.7
Iba1^+^/GFP^+^ cells	0.3 ± 0.3	0.3 ± 0.3	1.2 ± 0.6	0.7 ± 0.5	30.7 ± 4.6	10.0 ± 5.5*	15.2 ± 2.3*	9.7 ± 2.7**

The numbers of Iba1^+^ cells, GFP^+^ cells, Iba1^+^/GFP^–^ cells, Iba1^–^/GFP^+^ cells, and Iba1^+^/GFP^+^ cells were counted in the contralateral and ipsilateral spinal dorsal horn 14 days after pSNL surgery. “Total cells” indicates the sum of Iba1^+^/GFP^–^ cells, Iba1^–^/GFP^+^ cells, and Iba1^+^/GFP^+^ cells. *n* = 3–6. Data are expressed as mean ± SEM.

The number of Iba1^+^ cells and GFP^+^ cells dramatically increased in the ipsilateral dorsal horn, and 50–95% of GFP^+^ cells were double positive for Iba1. The number of Iba1^+^ cells decreased in TRPM2^BM–/Rec+^, TRPM2^BM+/Rec–^, and TRPM2^BM–/Rec–^ mice compared with that in TRPM2^BM+/Rec+^ mice, although no statistical difference was observed (*F*_3,13_ = 2.52, *p* = 0.104). Similarly, the number of GFP^+^ cells decreased in TRPM2^BM–/Rec+^, TRPM2^BM+/Rec–^ and TRPM2^BM–/Rec–^ mice, compared with that in TRPM2^BM+/Rec+^ mice, although no statistical difference was observed (*F*_3,13_ = 2.92, *p* = 0.074). Merged photographs revealed that the number of Iba1^+^/GFP^+^ cells significantly decreased in chimeric mice (*F*_3,13_ = 6.73, *p*<0.01). Significant decreases were observed in TRPM2^BM–/Rec+^, TRPM2^BM+/Rec–^, and TRPM2^BM–/Rec–^ mice compared with TRPM2^BM+/Rec+^ chimeric mice. However, no difference was observed in the numbers of Iba1^+^/GFP^–^ cells (*F*_3,13_ = 0.58, *p* = 0.641) or Iba1^–^/GFP^+^ cells (*F*_3,13_ = 0.25, *p* = 0.857) among chimera groups.

## Discussion

The results of the present study showed that pSNL-induced mechanical allodynia was attenuated by TRPM2 deficiency in either donor BM cells or recipient mice, similarly to that in TRPM2^BM–/Rec–^ mice. TRPM2 deficiency in either the donor BM cells or recipient mice led to reduced infiltration of GFP^+^ peripheral immune cells (particularly Iba1^+^ macrophages) into the dorsal horn. However, neither the infiltration of GFP^+^ peripheral immune cells (including Iba1^+^ macrophages) into the sciatic nerve, nor the number of Iba1^+^/GFP^–^ resident microglia in the spinal dorsal horn, were affected 14 days after pSNL surgery. These findings suggest that TRPM2 expressed not only in peripheral immune cells but also other cells such as spinal microglia plays an important role in the spinal infiltration of macrophages following peripheral nerve injury, which may contribute to neuropathic pain.

Our previous report [24] shows that TRPM2 deficiency reduces peripheral nerve injury-induced production of CXCL2, the most potent chemoattractant for neutrophils, which is produced by monocytes/macrophages [32]–[34], and that infiltration of neutrophils, but not macrophages, into the injured sciatic nerve. Furthermore, mechanical allodynia evoked by intraplantar injection of lipopolysaccharide (LPS)-stimulated macrophages was weak in TRPM2-KO-derived macrophages. However, these TRPM2-mediated responses of macrophages, *i.e.* CXCL2 production and neutrophil infiltration, in the injured sciatic nerve were partial, and restricted to the early phase of neuropathic pain (within 1 day). By contrast, the activation of spinal Iba1-or OX42-positive cells (referred to as microglia in the previous report) was suppressed in TRPM2-KO mice throughout the period of neuropathic pain. Therefore, although both peripheral macrophages and spinal microglia could be involved in neuropathic pain, it is possible that the prolonged inhibition of spinal microglia caused by TRPM2 deficiency may largely contribute to the prevention of neuropathic pain. This hypothesis is consistent with the present results, in which TRPM2^BM+/Rec–^ mice showed less pSNL-induced mechanical allodynia. However, the present data also showed that mechanical allodynia was attenuated in TRPM2^BM–/Rec+^ mice, suggesting the involvement of TRPM2 expressed by BM-derived peripheral immune cells including macrophages in neuropathic pain.

In the present chimeric mice, a large number of GFP^+^ peripheral immune cells were observed in the injured sciatic nerve, and more than half of them were Iba1^+^ macrophages. Consistent with our previous report [24], the number of Iba1^+^/GFP^+^ macrophages was not changed in any on the TRPM2-KO chimeric mice 14 days after pSNL surgery, suggesting that TRPM2 is not involved in the chemotactic activity of macrophages directed to the injured site. Additionally, the chimeric mice showed no effect on the infiltration of Iba1^–^/GFP^+^ peripheral immune cells other than macrophages, which may include neutrophils. Our previous report showed that TRPM2 deficiency reduced the infiltration of neutrophils into the injured sciatic nerve [24], probably due to decreased CXCL2 production by the resident and recruited macrophages. However, the peak time of the neutrophil infiltration was 1 day, and a low number of neutrophils were observed 14 days after pSNL surgery. Because the reduction of neutrophil infiltration by TRPM2 deficiency was observed only 8 h after pSNL surgery [24], it is possible that the reduction was not detected at 14 days after the surgery in the present study, although it has not been determined whether Iba1^–^/GFP^+^ peripheral immune cells, particularly Gr-1^+^ neutrophils, are reduced in TRPM2-KO BM chimeric mice 8 h after pSNL surgery.

By contrast, the number of Iba1^+^ cells in the spinal dorsal horn tended to decrease in all TRPM2-KO chimeric mice 14 days after pSNL surgery. However, the number of GFP^–^/Iba1^+^ cells, representing spinal-resident microglia, was not changed in any TRPM2-KO chimeric mice. These findings suggest that the increased number of Iba1^+^ cells in the spinal dorsal horn is mainly due to the increase in the spinal infiltration of Iba1^+^ macrophages, and that TRPM2 plays no role in the increased number of spinal-resident microglia, at least at 14 days after pSNL surgery. Notably, the increased number of GFP^–^/Iba1^+^ resident microglia represents an increase in microglial proliferation and/or chemotaxis from other regions, rather than microglial activation. The activation of spinal microglia (represented by an increase in Iba1 immunoreactivity and morphological changes to activation state) peaks at 3 days and declines within 2 weeks after pSNL surgery [24], while spinal infiltration of peripheral immune cells is delayed following the activation of spinal-resident microglia. Spinal infiltration by peripheral immune cells starts at 3 days and peaks at 14 days after peripheral nerve injury [6], [35]. Our previous report demonstrated that TRPM2 plays a critical role in the initial activation of spinal-resident microglia [24].

The present results showed that the number of GFP^+^ cells in the spinal dorsal horn, which represent infiltrated peripheral immune cells, tended to decrease in all TRPM2 chimeric mice. Furthermore, most infiltrated GFP^+^ peripheral immune cells are Iba1^+^ macrophages, which were markedly decreased in TRPM2 chimeric mice. Consistent with the present results, recent evidence suggests that peripheral nerve injury induces the infiltration of peripheral immune cells into the spinal cord [4]–[8]. The blood-spinal cord barrier (BSCB) constitutes a physical and biochemical barrier between the spinal cord and the peripheral circulation. Peripheral nerve injury triggers the leakage of the BSCB through spinal inflammatory responses, resulting the influx of inflammatory mediators and the infiltration of peripheral immune cells [35], [36]. Because spinally-infiltrated macrophages were differentiated as fully functional microglia, activation of both spinally-infiltrated macrophages and spinal-resident microglia contributes to the induction and persistence of neuropathic pain [6]. Taken together, these results suggest that inhibition of neuropathic pain observed in TRPM2-KO chimeric mice is due to the reduction of TRPM2-mediated spinal infiltration of macrophages, as well as activation of spinal-resident microglia. When using BM chimeric mice to study conditions of the central nervous system (CNS), some limitations should be considered. Whole-body irradiation has been reported to have direct consequences on the CNS, such as disruption of the blood-brain barrier [37]. Although we cannot ignore the effect of irradiation, an indisputable body of evidence suggests that disruption of BSCB and spinal infiltration of peripheral immune cells clearly occurs following peripheral nerve injury even in non-irradiated animals [5], [7], [8], [35], [38].

The mechanism underlying TRPM2-mediated spinal infiltration of macrophages is still unknown. As described above, TRPM2 plays no role in the chemotactic activity of macrophages. By contrast, TRPM2 deficiency attenuates peripheral nerve injury-induced activation of resident microglia, which precedes the spinal infiltration of macrophages. Consequently, TRPM2 deficiency may conceivably attenuate the initial deterioration of the spinal microenvironment by activating spinal-resident microglia, resulting in protection against disruption of the BSCB. However, leakage of the BSCB is not affected by intrathecal injection of minocycline, a microglial inhibitor, suggesting that BSCB disruption is independent of the activation of spinal-resident microglia [35]. Further investigations will be needed to elucidate the mechanisms.

### Conclusions

In summary, the present study revealed that TRPM2 expressed in peripheral immune cells and/or other cells plays a role in the spinal infiltration of macrophages, rather than infiltration of peripheral immune cells into the injured nerves and activation of spinal-resident microglia by using a set of WT/TRPM2-KO BM chimeric mice. Furthermore, the spinal infiltration of macrophages mediated through TRPM2 is suggested to contribute to the induction and persistence of neuropathic pain. The present findings provide evidence for a role of TRPM2 in neuropathic pain, suggesting that TRPM2 might be a promising target for the treatment of neuropathic pain.
